# Risk factor of pneumonitis resulting from thoracic chemoradiotherapy combined with immunotherapy in lung cancer: A meta-analysis and systematic review

**DOI:** 10.1016/j.isci.2025.113761

**Published:** 2025-10-25

**Authors:** Yingding Ruan, Jianwei Han, Wenjun Cao, Chuan Long, Aiming Yang, Zhendong Chen, Siyu Guo, Ting Zhang

**Affiliations:** 1 Department of Thoracic Surgery, The First People’s Hospital of Jiande, Jiande 311600, China; 2 Department of Thoracic Surgery, Affiliated Zhongshan Hospital of Dalian University, Dalian 116001, China; 3 Department of Oncology, The First People’s Hospital of Jiande, Jiande 311600, China; 4 Radiotherapy Department, Second Affiliated Hospital, Zhejiang University School of Medicine, Hangzhou 310009, China

**Keywords:** oncology, therapeutics

## Abstract

This study was a systematic review and meta-analysis of the risk factors associated with pneumonitis resulting from the combination of thoracic chemoradiotherapy (CRT) and immunotherapy in patients with lung cancer. A comprehensive search was conducted on PubMed, Web of Science, ScienceDirect, and Cochrane Library databases to identify clinical studies that investigated the risk factors for pneumonitis in patients with lung cancer undergoing CRT combined with immunotherapy. The study adhered to the Preferred Reporting Items for Systematic Reviews and Meta-Analyses (PRISMA) guidelines and checklists. We analyzed 17 studies. Our comprehensive analysis identified volume (V) 20 and V40 as significant risk factors for pneumonitis in patients with lung cancer receiving CRT combined with immunotherapy (hazard ratio (HR) = 1.03, 95% confidence interval (95% CI) = 1.00–1.06). Additionally, body mass index ≥30 was a risk factor in a subset analysis (HR = 2.24, 95% CI = 1.12–4.48). In subset analyses, individuals aged ≥65 years had a higher risk of pneumonitis compared with those aged <65 years (HR = 1.39, 95% CI = 1.08–1.80), but this association was not significant when all age data were pooled (HR = 1.02, 95% CI = 0.99–1.05). Smoking was inversely associated with the risk of pneumonitis (HR = 0.93, 95% CI = 0.88–0.97). Race did not significantly affect the risk of pneumonitis, but whites had a lower risk compared with other races in subset es (HR = 0.78, 95% CI = 0.62–0.97). This meta-analysis and systematic review revealed that V20, V40, and body mass index ≥30 were significant risk factors for pneumonitis in patients with lung cancer receiving CRT combined with immunotherapy. Older patients also had a higher risk. Smoking history, however, was inversely associated with the risk of pneumonitis.

## Highlights

• V20, V40, and BMI ≥30 are key pneumonitis risk factors in lung cancer CRT+ICI
• Older age (≥65) increases pneumonitis risk; smoking shows an inverse link
• Sex, TNM stage, PD-L1, ECOG, and COPD show no significant link to pneumonitis
• Optimized radiotherapy and monitoring are essential to manage pneumonitis in CRT+ICI

## Graphical abstract

**Figure:**
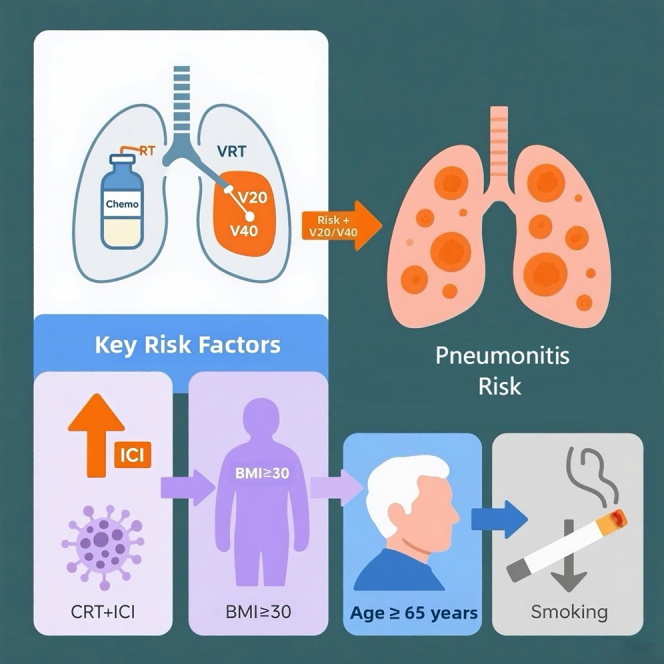

## Introduction

Lung cancer remains one of the leading causes of mortality globally.^1^^,^^2^ The advent of immune checkpoint inhibitors (ICIs) has marked a significant milestone in lung cancer treatment, with various immunotherapies demonstrating promising results in advanced non-small cell lung cancer (NSCLC), significantly enhancing overall response and survival rates.^3^^,^^4^^,^^5^ Recent preclinical and clinical evidence has further shown that the combination of chemoradiotherapy (CRT) and ICIs exhibits synergistic antitumor effects.^6^^,^^7^^,^^8^^,^^9^ Notably, the PACIFIC trial has underscored the benefits of this combination, revealing that the use of a programmed death-ligand 1 (PD-L1) inhibitor alongside CRT in unresectable stage III NSCLC led to an improvement in overall survival (OS) rates, with a 5-year OS rate as high as 42.9% when durvalumab was used as consolidative therapy following CRT.^8^^,^^10^ For patients with CRT without disease progression, durvalumab administration for up to 12 months has become the new standard of care.^11^ The combination of immunotherapy and chemotherapy has emerged as the standard treatment regimen for advanced NSCLC, and for patients with oligometastatic disease, the incorporation of radiotherapy can further enhance survival. The early RTOG 0937 study was a randomized phase II trial focused on consolidative radiation therapy following chemotherapy in patients with extensive-stage small cell lung cancer (SCLC).^12^ Subsequent to RTOG 0937, further research, such as the Astrum-LC01 study in limited-stage SCLC,^13^ demonstrated the promising therapeutic effects of combining radiotherapy with immunotherapy across different stages and types of lung cancer. These findings established this combined approach as a standard treatment option.

The mechanism of action of ICIs involves blocking immune checkpoints, such as programmed cell death protein 1 (PD-1)/PD-L1 and cytotoxic T-lymphocyte-associated antigen (CTLA)-4, thereby restoring the function of immune cells to attack cancer cells. However, enhanced immune activation can also attack healthy tissues, leading to adverse events, including pneumonitis.^14^^,^^15^ When combined with chemotherapy and radiotherapy, ICIs can potentially yield a synergistic therapeutic effect, exhibiting a synergistic interaction that surpasses additive effects in terms of efficacy.^16^^,^^17^^,^^18^ However, the safety profile of this combined regimen remains a concern, as demonstrated by a study that reported an incidence of grade ≥3 pneumonitis has increased in patients undergoing thoracic chemoradiotherapy with immunotherapy.^19^ Given the significant adverse effects and risks associated with this treatment combination, identifying the relevant risk factors for pneumonitis is crucial for refining and optimizing strategies to better manage and control this condition in patients with lung cancer.

Several clinical trials have investigated the efficacy and safety of concurrent chemoradiotherapy combined with ICI (CRT+ICI).^20^^,^^21^^,^^22^ However, further research is needed to determine whether this combination increases the risk of pneumonitis. Currently, an increasing amount of data is available from studies on the use of CRT+ICI for the treatment of lung cancer. Multiple studies have explored various potential risk factors for pneumonitis in lung cancer populations undergoing this combined therapy.^23^^,^^24^^,^^25^^,^^26^^,^^27^^,^^28^^,^^29^^,^^30^^,^^31^^,^^32^^,^^33^^,^^34^^,^^35^^,^^36^^,^^37^^,^^38^^,^^39^ However, the research findings are inconsistent and often limited by small sample sizes, methodological differences, and potential biases. We conducted a systematic review and meta-analysis of CRT combined with immunotherapy in patients with lung cancer to explore the risk factors for pneumonitis induced by this combined treatment approach.

### Methods

Following the Preferred Reporting Items for Systematic Reviews and Meta-Analyses (PRISMA)2020 guidelines,^40^ we performed a systematic review and meta-analysis with methodological. The analysis protocol was prospectively registered in the PROSPERO International Register of Systematic Reviews (CRD42024612910). Key procedures included systematic literature searches, dual independent review for study inclusion, and standardized data synthesis to ensure analytical consistency.

### Search strategy

A comprehensive literature search was conducted between July 2024 and October 2024 by two independent investigators in Cochrane Library, ScienceDirect, Web of Science, and PubMed. The search terms used across all databases were: (“Pneumonitis” OR “Radiation Pneumonitis” OR “Chemotherapy-induced Pneumonitis”) AND (“Chemoradiation” OR “Chemoradiotherapy” OR “Concurrent Chemoradiation Therapy”) AND (“Risk Factors” OR “Risk Factor”) AND (“Lung Cancer” OR “NSCLC” OR “Small Cell Lung Cancer”).

### Inclusion and exclusion criteria

Randomized controlled trials (RCTs) and nonrandomized controlled studies were both eligible for our analysis. The inclusion criteria were: (1) patients with a histologically or cytologically confirmed diagnosis of lung cancer; (2) patients who received either thoracic CRT combined with immunotherapy, immunotherapy given concurrently with CRT, or consolidation immunotheraphy; and (3) English language articles. The exclusion criteria were: (1) case reports, commentaries, editorials, conference abstracts, or other nonoriginal research; (2) articles that did not clearly report or analyze risk factors for pneumonitis; (3) articles that were unavailable or inaccessible in full-text form; (4) studies with incomplete, obviously erroneous, or unusable data for analysis; and (5) studies with severe bias or quality problems, including but not limited to selection, information, and confounding bias.

A two-phase screening protocol was implemented by two independent reviewers (Jianwei Han and Yingding Ruan). The initial deduplication phase evaluated records based on title, publication year, and author metadata. Following duplicate removal, a secondary eligibility assessment was conducted by the same reviewers, systematically examining titles, abstracts, and full texts. Discrepancies at any stage were resolved through third-party adjudication (Ting Zhang) to ensure consensus.

### Data extraction

Data extraction from eligible studies was performed by two independent reviewers (Jianwei Han and Yingding Ruan). The extracted data included the first author’s name, publication year, country of origin, study type, type of lung tumor, treatment categories, treatment sequence, total patient count, number of patients receiving CRT+ICI, pneumonitis grade, and number of patients with pneumonitis in the CRT+ICI group. Data also included demographic and clinical characteristics: sex; age; body mass index (BMI); smoking status; history of chronic pulmonary diseases [interstitial lung disease (ILD), chronic obstructive pulmonary disease (COPD), asthma, or tuberculosis (TB)]; PD-L1 expression; tumor, node, and metastasis (TNM) stage; and Eastern Cooperative Oncology Group (ECOG) score.

We gathered odds ratio (OR) and hazard ratio (HR) values, along with their 95% confidence intervals (CIs), from individual studies for univariable and multivariable analyses of pneumonitis grades ≥1 or ≥2. The data were stratified by sex, age, BMI, smoking history, chronic pulmonary disease status, PD-L1 expression, TNM stage, ECOG performance status (PS), and race. The lung dose-volume parameters were also recorded, including percent volume of the lung receiving ≥5 Gy (V5), percent volume of lung receiving ≥20 Gy (V20), percent volume of lung receiving ≥40 Gy (V40), mean lung dose (MLD), and total lung volume (TLV). OR and HR signified the risk of developing pneumonitis. Pneumonitis grade was assessed using the Common Terminology Criteria for Adverse Events version 5.0 (or 4.0) grading system (Table 1). Any discrepancies were resolved by a third investigator (Ting Zhang) to guarantee accuracy and consistency.

**Table 1:** Common terminology criteria for adverse events (CTCAE) version 5.0 (4.0) grading system

Grade1	Asymptomatic; clinical or diagnostic observations only; intervention not indicated
Grade2	Symptomatic; medical intervention indicated; limiting instrumental ADL;
Grade3	Severe symptoms; limiting self-care ADL; oxygen indicated;
Grade4	Life-threatening respiratory compromise; urgent intervention indicated (e.g., tracheotomy or intubation)
Grade5	Death

ADL, Activities of Daily Living.

### Assessment of bias

Study quality was evaluated using the Newcastle-Ottawa Scale (NOS), a validated tool recommended by the Cochrane Collaboration for assessing non-randomized studies.^41^ The NOS framework comprises three domains: population selection (max 4 points), cohort comparability (max 2 points), and outcome/exposure ascertainment (max 3 points), with a total possible score of 9. Following contemporary practice,^42^ studies achieving ≥5 points were classified as moderate-to-high quality. Dual independent appraisers (Jianwei Han and Yingding Ruan) conducted quality assessments using this structured instrument. Inter-rater discrepancies were resolved through consensus discussions moderated by a third reviewer (Ting Zhang), ensuring the standardized interpretation of methodological rigor.

### Statistical analysis

We conducted an analysis focusing on patients with pneumonitis grade ≥2. The meta-analysis was conducted using Stata 12.0 and Review Manager 5.4.1. Using Stata 12.0, we converted HR, OR, and their corresponding 95% CIs into log(HR) and standard error values. Subsequent analyses were conducted in Review Manager 5.4.1. Heterogeneity was assessed using two complementary methods: the I^2^ statistic for quantifying inconsistency and Cochrane’s Q test (α = 0.10) for qualitative evaluation. The analytic framework was determined by heterogeneity thresholds: Fixed-effects models were used if heterogeneity was low (I^2^ ≤ 50%, *p* ≥ 0.10), whereas random-effects models were employed for substantial heterogeneity (I^2^ >50%, *p* < 0.10). For methodologically heterogeneous variables, random-effects models were applied irrespective of thresholds to account for institutional variations in dose protocols. This balances statistical rigor with clinical realism in observational meta-analyses.

Funnel plot symmetry and non-significant statistical thresholds (all *p* > 0.05) jointly suggested minimal publication bias risk. To evaluate the robustness of findings, sensitivity analyses were conducted. Sensitivity analyses confirmed result stability, as effect estimates consistently fell within their 95% CIs. Reproducibility was further validated by comparable outcomes after sequentially excluding each study.

## Results

### Characteristics of the included studies

**Figure 1:**
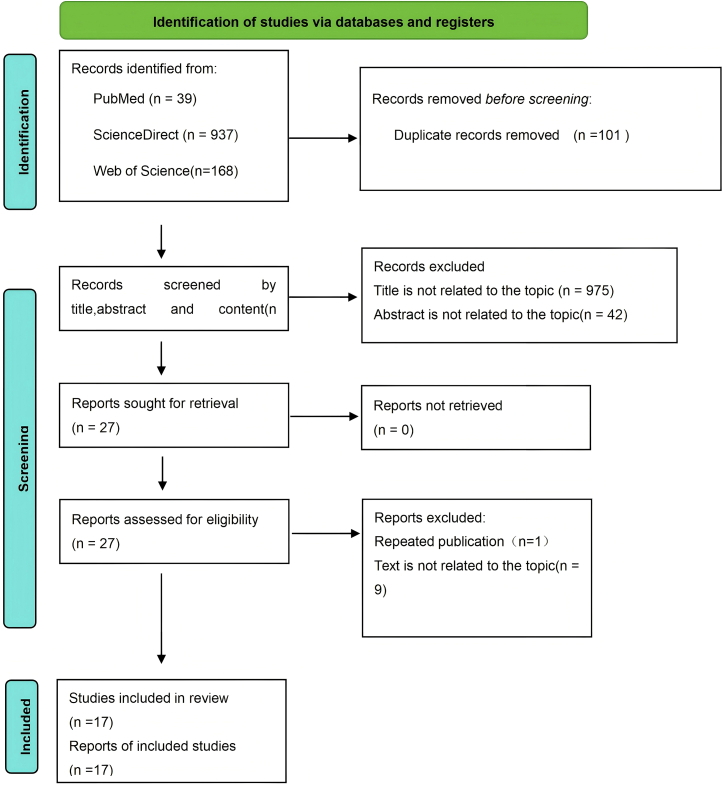
The PRISMA flow diagram displays the details of the selection process ∗*From*: Page, M.J., McKenzie, J.E., Bossuyt, P.M., Boutron, I., Hofmann, T.C., Mulrow, C.D., Shamseer, L., Tetzlaf, J.M., Akl, E.A., Brennan, S.E. et al. (2021). The PRISMA 2020 statement: an updated guideline for reporting systematic reviews. Syst Rev. Mar 29;10(1):89. https://doi.org/10.1186/s13643-021-01626-4. PMID: 33781348; PMCID: PMC8008539.

The PRISMA flow diagram (Figure 1) shows the details of the selection process. A total of 1112 records were identified from the four databases. Following title and abstract screening, 23 records underwent full-text review. Six articles were excluded because of a lack of data on pneumonitis. Of the 17 studies^23^^,^^24^^,^^25^^,^^26^^,^^27^^,^^28^^,^^29^^,^^30^^,^^31^^,^^32^^,^^33^^,^^34^^,^^35^^,^^36^^,^^37^^,^^38^^,^^39^ that met the selection criteria, seven were multicenter^27^^,^^29^^,^^31^^,^^35^^,^^37^^,^^38^^,^^39^ and 10 were single center^23^^,^^24^^,^^25^^,^^26^^,^^28^^,^^30^^,^^32^^,^^33^^,^^34^^,^^36^; all of which were of moderate-to-high quality (Table 2). Characteristics of the included studies are shown in Table 3.

**Table 2:** The Newcastle-Ottawa scale (NOS) for assessing the quality of nonrandomized studies in our study

Study	Year	Country	Type of Article	The Newcastle-Ottawa Scale (NOS)		
				Selection	Comparability	Exposure
Jang et al.^23^	2021	Korea	Single-center, retrospective study	∗ ∗ ∗ ∗	∗ ∗	∗ ∗ ∗
Altan et al.^24^	2023	USA	Single-center, retrospective study	∗ ∗ ∗ ∗	∗ ∗	∗ ∗ ∗
Akkad et al.^25^	2023	USA	Retrospective,a real-world study	∗ ∗ ∗ ∗	∗ ∗	∗ ∗ ∗
Dan Pu et al.^26^	2024	China	Single-center, retrospective study	∗ ∗ ∗ ∗	∗ ∗	∗ ∗ ∗
Vansteenkiste et al.^27^	2024	Belgium	Phase 3 PACIFIC, trial,randomized, double-blind trial	∗ ∗ ∗ ∗	∗ ∗	∗ ∗ ∗
Mayahara et al.^28^	2022	Japan	Single-center, retrospective study	∗ ∗ ∗ ∗	∗	∗ ∗
Yegya-Raman et al.^29^	2023	USA	Single institution, multisite retrospective study	∗ ∗ ∗ ∗	∗ ∗	∗ ∗ ∗
Sato et al.^30^	2024	Japan	Single-center, retrospective study	∗ ∗ ∗ ∗	∗ ∗	∗ ∗ ∗
Lim et al.^31^	2023	Canada	Two-centers, a real-world populaiton, retrospective study	∗ ∗ ∗	∗ ∗	∗ ∗ ∗
Gao et al.^32^	2022	USA	Single-center, retrospective study	∗ ∗ ∗	∗	∗ ∗
Harada et al.^33^	2021	Japan	Single-center, retrospective study	∗ ∗ ∗	∗	∗ ∗
Diamond et al.^34^	2023	USA	Single-center, retrospective study	∗ ∗ ∗ ∗	∗	∗ ∗
Shintani et al.^35^	2021	Japan	Multi-institutional retrospective study	∗ ∗ ∗ ∗	∗ ∗	∗ ∗ ∗
Portal et al.^36^	2023	USA	Single-center, retrospective study	∗ ∗ ∗ ∗	∗ ∗	∗ ∗ ∗
Tsukita et al.^37^	2021	Japan	Multicenter, retrospective, observational, study	∗ ∗ ∗ ∗	∗ ∗	∗ ∗ ∗
Edwards et al.^38^	2024	USA	A retrospective study, national Veterans Health Administration	∗ ∗ ∗ ∗	∗ ∗	∗ ∗ ∗
Park et al.^39^	2023	Korean	Multicenter, retrospective, observational study, PACIFIC-KR trial	∗ ∗ ∗ ∗	∗ ∗	∗ ∗ ∗

∗:1 point.

**Table 3:** Patients, characteristics, pathology, pneumonitis, and treatment outcomes summary

Authors	Year	Country	Type of Article	Type of Tumor	Categories	Treated	Total (n)	CRT+ICI(n)	Pneumonitis grade	Pneumonitis in CRT+ICI (n)	Age (Y) (M(IQR))	Male (n,%)	BMI (kg/m^2^) (M(IQR)	Smoking (n,%)	History of chronic pulmonary disease (n,%)				Expression PD-L1 IHC (n,%)			TNM stage (n,%)				ECOG PS (n,%)			
															ILD	COPD	Asthma	TB	TPS<1%	1%≤TPS≤49%	TPS≥50%	I	Ⅱ	Ⅲ	Ⅳ	0	1	2	3
Jang et al.^20^	2021	Korea	Single-center, retrospective study	NSCLC	CRT first	CRT after ICI	106	51	≥ Grade 2	27	64 (38–82)	89 (84.0)	–	82 (77.4)	1 (0.9)	13 (12.3)	4 (3.8)	8 (7.5)	8 (15.7)	36 (70.6)		–	10 (9.4)	96 (90.6)	–	45 (88.2)		3 (5.9)	–
Altan et al.^21^	2023	USA	Single-center, retrospective study	LA-NSCLC	Concurrent	CRT with D	140	150	≥ Grade 2	32	67 (61–72)	74 (53.0)	–	121 (87)	7 (5)	39 (27)	–	–	–			–				–			
Akkad et al.^22^	2023	USA	Retrospective,a real-world study,	LA-NSCLC	CRT first	CRT after D	284	284	≥ Grade 2	61	68 (39–88)	271 (96.0)	26 (M)	–	–				39 (14)	41 (14)	32 (11)	1 (<1)	21 (7)	228 (80)	–	–			
Dan Pu et al.^23^	2024	China	Single-center, retrospective study	Lung cancer	CRT first	CRT after ICI	152	58	≥ Grade 2	23	59 (54–66)	129 (84.9)	–	68 (44.7)	19 (12.5)				–			–	–	111 (73)	41 (27)	–			
									≥ Grade 3	9																			
Vansteenkiste et al.^24^	2024	Belgium	phase 3 PACIFIC trial, randomized, double-blind trial	unresectable stage III NSCLC	CRT first	CRT after D	713	475	≥ Grade 2	94	–	–	–	–	–	93 (20.3)	–	–	–			–				–			
Mayahara et al.^25^	2022	Japan	Single-center, retrospective study	LA-NSCLC	CRT first	CRT after D	56	56	≥ Grade 2	40	72 (48–85)	37 (66.0)	–	45 (80)	–				19 (24)	11 (20)	9 (16)	–	–	43 (77)	–	24 (43)	28 (50)	4 (7)	–
Yegya-Raman et al.^26^	2023	USA	single institution, multisite retrospective study	LA-NSCLC	CRT first	CRT after ICI	783	335	≥ Grade 2	108	68 (62–74)$	155 (46.0)	27.2 (23.8–30.9)	–	–				–			–	23 (7)	313 (93)	–	115 (34)	185 (55)	35(11)	0 (0)
Sato et al.^27^	2024	Japan	Single-center, retrospective study	Stage III NSCLC	CRT first	CRT after D	150	81	≥ Grade 2	28	71 (44–84)$	48 (70.0)	–	60 (97)	–				15 (22)	19 (28)	13 (19)	–	–	69 (100)	–	33 (48)	32 (46)	4(6)	–
Lim et al.^28^	2023	Canada	Two-centers, a real-world populaiton, retrospective study	LA-NSCLC	CRT first	CRT after D	189	189	≥ Grade 1	49	67 (30–84)	88 (47.0)	–	177 (94)	–				36 (19)	45 (24)	59 (31)	–				40 (21)	132 (70)	17 (9)	
Gao et al.^29^	2022	USA	Single-center, retrospective study	LA-NSCLC	CRT first	CRT after D	190	190	≥ Grade 2	50	67 (61–75)	93 (48.9)	–	–	–	71 (37.8)	–	–	–			7 (3.7)	11 (5.8)	171 (90)	1 (0.5)	78 (41.1)	88 (46.3)	24 (12.6)	–
Harada et al.^30^	2021	Japan	Single-center, retrospective study	LA-NSCLC	CRT first	CRT after D	26	26	≥ Grade 2	10	65.5 (43–77)	20 (77.0)	–	23 (88)	–	12 (46)	–	–	4 (15)	8(31)	12 (46)	–	–	36 (100)	–	6 (23)	20 (77)	–	–
Diamond et al.^31^	2023	USA	Single-center, retrospective study	LA-NSCLC	CRT first	CRT after D	62	62	≥ Grade 2	20	67 (62–73)	30 (48.0)	–	62 (100)	–	26 (42)	–	–	–			–	6 (10)	56 (90)	–	–			
Shintani et al.^32^	2021	Japan	multi-institutional retrospective study	NSCLC	CRT first	CRT after D	146	146	≥ Grade 2	50	70 (63–75)	120 (82.2)	–	126 (86.3)	–				33 (22.6)	38 (26)	43 (29.5)	–	7 (4.8)	126 (86.3)	1 (0.7)	85 (58.2)	54(37)	6 (4.1)	–
Portal et al.^33^	2023	USA	Single-center, retrospective study	LA-NSCLC	CRT first	CRT Concurrent/Sequential ICI	25	25	≥ Grade 1	5	74 (51–83)	12 (48.0)	27.08 (26.93–44.19)	22 (88)	–				–			–	1 (4)	24 (96)	–	7 (28)	17 (68)	1 (4)	–
Tsukita et al.^34^	2021	Japan	Multicenter, retrospective, observational, study	Stage III NSCLC	CRT first	CRT after D	107	87	≥ Grade 2	38	70 (43–86)	76 (71.0)	–	92 (86)	–				22 (20.6)	29 (27.1)	31 (29)	–	–	107 (100)	–	71 (66.4)	36 (33.6)	0 (0)	–
Edwards et al.^35^	2024	USA	A retrospective study, national Veterans Health Administration	Stage III NSCLC	CRT first	CRT after D	1994	1005	≥ Grade 2	210	69 (64,72)	958 (95.0)	–	837 (83)	–	518 (52)	–	–	–			–	–	1994 (100)	–	–			
Park et al.^36^	2023	Korean	Multicenter, retrospective, observational study, PACIFIC-KR trial	Stage III NSCLC	CRT first	CRT after D	157	157	≥ Grade 2	57	65 (36–82)	134 (85.4)	–	126 (80.3)	1 (0.6)	50 (31.8)	–	–	19 (12.1)	72 (45.9)	52 (33.1)	–	–	157 (100)	–	41 (27.4)	97 (61.8)	14 (8.9)	3 (1.9)

M, median; NSCLC, non-small-cell lung cancer; LA-NSCLC, locally advanced non-small cell lung cancer; ICI, immune checkpoint inhibitor; CRT: chemoradiotherapy; ILD: interstitial lung disease; COPD, chronic obstructive pulmonary disease; TB: tuberculosis; D, durvalumab; PD-L1, programmed cell death ligand 1; Y, years; BMI, body mass index; ECOG PS, Eastern Cooperative Oncology Group Performance Status; IQR, interquartile range;IHC, immunohistochemistry; TPS, tissue polypeptide specific antigen; TNM stage, the tumor, node, and metastasis stage. $: Represents the data results of the pneumonitis group.

A total of 5280 patients were included in the analysis, with 3367 patients with lung cancer receiving CRT + ICI. Among these, 911 patients developed pneumonitis during or after CRT+ICI treatment. All studies conducted both univariate and multivariate analyses on patients who developed pneumonitis during or after CRT+ICI. Fifteen studies analyzed patients with pneumonitis grade ≥2,^23^^,^^24^^,^^25^^,^^26^^,^^27^^,^^28^^,^^29^^,^^30^^,^^32^^,^^33^^,^^34^^,^^35^^,^^37^^,^^38^^,^^39^ two studies analyzed grade ≥1,^31^^,^^36^ and one study analyzed grade ≥3.^26^ Additionally, thirteen studies reported HR,^23^^,^^24^^,^^28^^,^^29^^,^^30^^,^^31^^,^^32^^,^^33^^,^^34^^,^^35^^,^^36^^,^^38^^,^^39^ while four studies reported OR.^25^^,^^26^^,^^27^^,^^37^ Twelve studies reported RCT+durvalumab treatment.^24^^,^^25^^,^^27^^,^^28^^,^^30^^,^^31^^,^^32^^,^^33^^,^^34^^,^^35^^,^^37^^,^^38^^,^^39^ Four studies conducted both univariate and multivariate analyses on the overall incidence of pneumonitis without differentiating the causative treatment regimens.^36^^,^^37^^,^^38^^,^^39^

### Analysis of risk factors for pneumonitis following chemoradiotherapy+immune checkpoint inhibitors treatment

#### Sex

**Figure 2:**
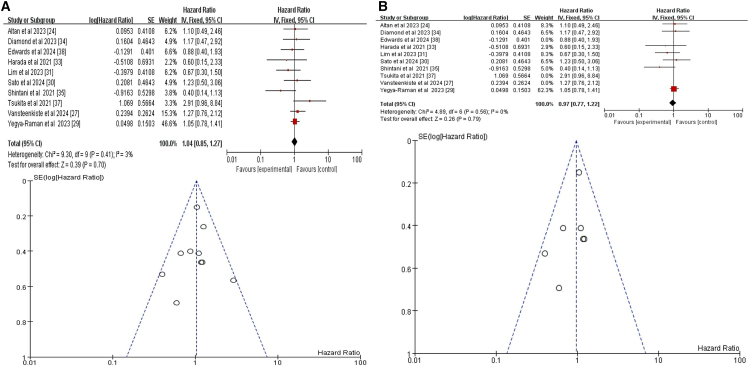
Forest plot and funnel plot of studies on sex and pneumonitis risk (A) Fixed-effects model for the risk of pneumonia related to sex. (B) Excluding three studies on treatment-unspecified pneumonitis, the fixed-effects model analyzed sex-related pneumonia risk. CI, confidence interval; HR, hazard ratio; RR, risk ratio; M-H, Mantel-Haenszel; SE, standard error.

Our initial meta-analysis of 10 studies showed that sex was not a significant predictor of CRT+ICI-induced pneumonitis (HR = 1.04, 95% CI = 0.85–1.27, *p* = 0.7)^24^^,^^27^^,^^29^^,^^30^^,^^31^^,^^33^^,^^34^^,^^35^^,^^37^^,^^38^ (Figure 2A). To further ensure accuracy, we excluded three studies that did not distinguish between pneumonitis caused by different treatment regimens.^27^^,^^37^^,^^38^ The refined meta-analysis of the remaining seven studies found no significant association between sex and CRT+ICI-induced pneumonitis risk (HR = 0.97, 95% CI = 0.77–1.22, *p* = 0.79) (Figure 2B). The consistency of our results before and after exclusions supports the robustness of our conclusion.

#### Age

**Figure 3:**
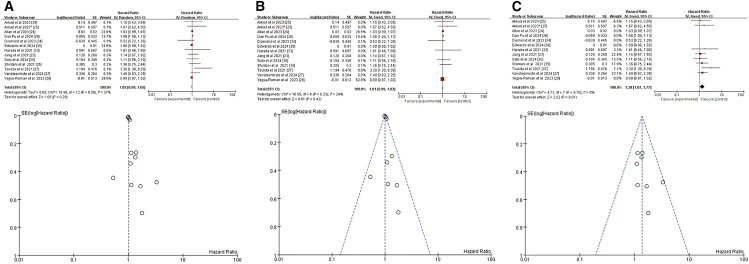
Forest plot and funnel plot of studies on age and pneumonitis risk (A) Fixed-effects model for the risk of pneumonia related to age. (B) Excluding four studies on treatment-unspecified pneumonitis, the fixed-effects model analyzed age-related pneumonia risk. (C) Fixed-effects model of studies restricted to patients aged ≥65 or ≥70 years for pneumonitis risk.

Initially, twelve studies were included, augmented by an additional dataset from one study, resulting in a total of thirteen datasets for analysis.^23^^,^^24^^,^^25^^,^^26^^,^^27^^,^^29^^,^^30^^,^^33^^,^^34^^,^^35^^,^^36^^,^^37^^,^^38^ Our initial meta-analysis of these thirteen datasets indicated that age was not a significant predictor of CRT+ICI-induced pneumonitis (HR = 1.02, 95% CI = 0.99–1.05, *p* = 0.29) (Figure 3A). To reduce the impact of confounding variables, we excluded four studies^23^^,^^27^^,^^37^^,^^38^ that did not differentiate causes of pneumonitis and reanalyzed the remaining eight studies (nine datasets); again finding no significant effect of age (HR = 1.01, 95% CI 0.99–1.03, *p* = 0.42) (Figure 3B). However, subgroup analysis of patients aged ≥65 or ≥70 years (seven studies, eight datasets) demonstrated a significant increase in the risk of pneumonitis (HR = 1.39, 95% CI = 1.08–1.80], *p* = 0.01) (Figure 3C),^23^^,^^25^^,^^27^^,^^30^^,^^33^^,^^35^^,^^37^ indicating greater susceptibility in older patients.

#### Body mass index

**Figure 4:**
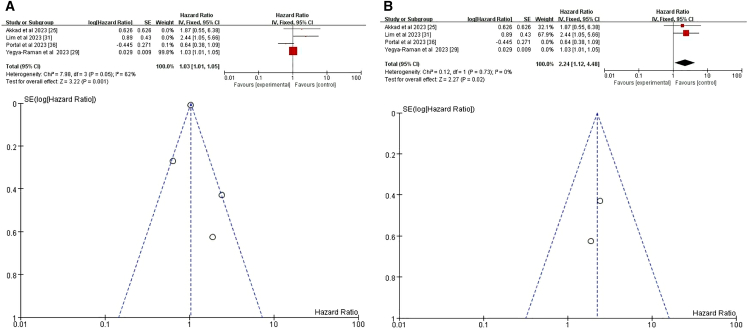
Forest plot and funnel plot of studies that BMI for pneumonitis risk (A) Random-effects model for the risk of pneumonia related to BMI. (B) Fixed-effects model of studies that BMI ≥30 kg/m^2^ for pneumonitis risk. BMI, body mass index.

We analyzed four relevant studies,^25^^,^^29^^,^^31^^,^^36^ all of which evaluated the relationship between BMI and pneumonitis following CRT+ICI. A weak but significant positive correlation between BMI and pneumonitis risk emerged (HR = 1.03, 95% CI = 1.01–1.05, *p* = 0.001) (Figure 4A). For a deeper analysis of the potential impact of BMI on pneumonitis risk and to minimize the interference of confounding factors, we focused on a subgroup analysis of patients with BMI ≥30. A meta-analysis of two studies specifically addressing BMI ≥30 showed that this threshold was a significant risk factor for pneumonitis following CRT+ICI (HR = 2.24, 95% CI = 1.12–4.48, *p* = 0.02) (Figure 4B).^25^^,^^31^ These findings indicate that BMI ≥30 significantly increases the risk of pneumonitis after CRT+ICI treatment.

#### Tumor, node, and metastasis stage

**Figure 5:**
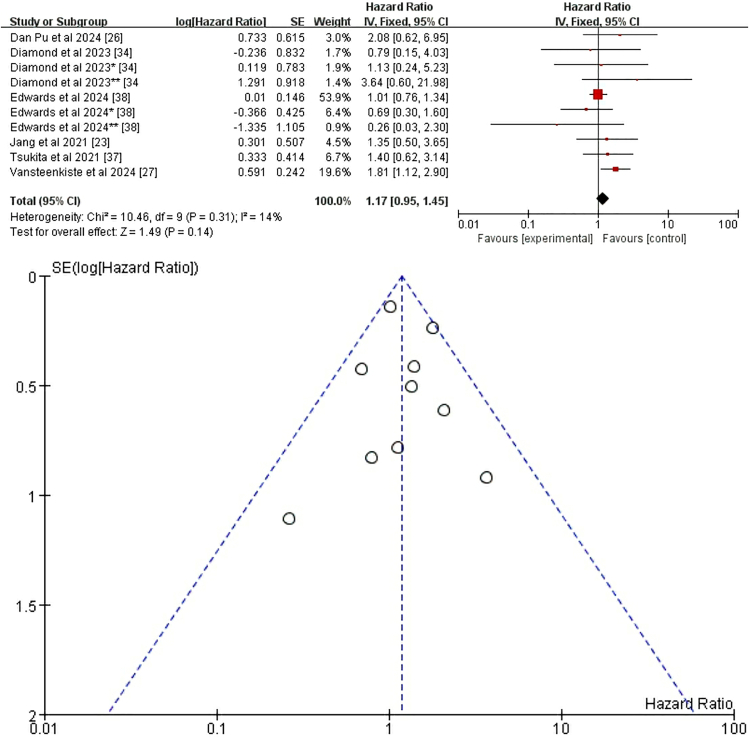
Fixed-effects model with forest plot and funnel plot for assessing the risk of pneumonia related to the TNM staging system TNM stage, the tumor, node, and metastasis stage.

Six studies explored the relationship between TNM status and CRT+ICI-induced pneumonitis.^23^^,^^26^^,^^27^^,^^34^^,^^37^^,^^38^ Two of these studies provided four subsets of age-related data,^34^^,^^38^ contributing to a comprehensive dataset comprising 10 groups for analysis. The selected studies exhibited variability in patient populations regarding specific TNM stages. Specifically, one study targeted locally advanced (LA)-NSCLC,^34^ two focused on stage III NSCLC,^37^^,^^38^ while another encompassed a lung cancer population.^26^ Additionally, one study concentrated on NSCLC,^23^ and another specifically addressed unresectable stage III NSCLC.^27^ Despite this variability, all studies examined patients with TNM stages beyond IIB, making it possible to draw conclusions pertinent to this higher-risk cohort. The pooled analysis data from these six studies indicated that, regardless of fixed-effects or random-effects models, there was no significant association between lung cancer stage >IIB and the incidence of pneumonitis related to CTR+ICI treatment (HR = 1.17, 95% CI = 0.95–1.45, *p* = 0.14) (Figure 5).

#### Smoking

**Figure 6:**
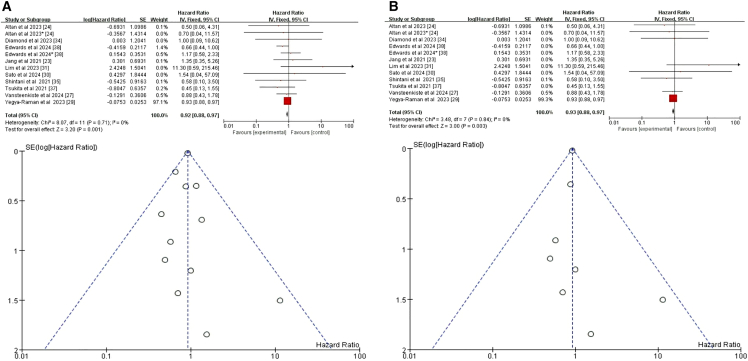
Forest plot and funnel plot of studies on smoking and pneumonitis risk (A) Fixed-effects model for the risk of pneumonia related to smoking. (B) Excluding four studies on treatment-unspecified pneumonitis, the fixed-effects model analyzed smoking-related pneumonia risk.

Among the seventeen studies included in our analysis, ten specifically addressed the relationship between smoking and CRT+ICI-induced pneumonitis.^23^^,^^24^^,^^27^^,^^29^^,^^30^^,^^31^^,^^34^^,^^35^^,^^37^^,^^38^ Notably, two of these studies contributed two separate datasets on smoking-related factors,^24^^,^^38^ resulting in twelve datasets for comprehensive analysis. The initial pooled analysis of data from all ten studies revealed a significant inverse association between smoking and risk of CRT+ICI-induced pneumonitis (HR = 0.92, 95% CI = 0.88–0.97, *p* = 0.001) (Figure 6A), suggesting smokers had a lower risk compared to nonsmokers. To refine the analysis and minimize confounding, we excluded three studies that did not distinguish treatment regimens causing pneumonitis.^23^^,^^37^^,^^38^ The revised analysis, based on six studies and seven datasets, confirmed consistent results (HR = 0.93, 95% CI = 0.88–0.97, *p* = 0.003) (Figure 6B).^24^^,^^27^^,^^29^^,^^30^^,^^31^^,^^34^^,^^35^ Both analyses robustly indicated smoking as a significant, albeit inversely related, risk factor for CRT+ICI-induced pneumonitis.

#### Race

**Figure 7:**
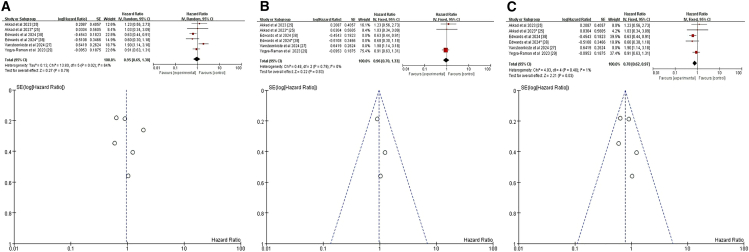
Forest plot and funnel plot of studies on race and pneumonitis risk (A) Random-effects model for the risk of pneumonia related to race. (B) Excluding two studies on treatment-unspecified pneumonitis, the fixed-effects model analyzed sex-related pneumonia risk. (C) Fixed-effects model of studies that included White for pneumonitis risk.

Among the seventeen studies reviewed, four specifically addressed the relationship between race and CRT+ICI-induced pneumonitis, with two studies contributing two datasets each, totaling six datasets for analysis.^25^^,^^27^^,^^29^^,^^38^ The initial pooled analysis of these four studies revealed no significant association between race and risk of CRT+ICI-induced pneumonitis (HR = 0.95, 95% CI = 0.65–1.38, *p* = 0.79) (Figure 7A). To refine the analysis and minimize confounding, we excluded two studies that did not distinguish between treatment regimens causing pneumonitis.^27^^,^^38^ The revised analysis, incorporating data from two studies and three datasets, yielded consistent results (HR = 0.96, 95% CI = 0.70–1.33], *p* = 0.83) (Figure 7B).^25^^,^^29^ However, a subanalysis focusing on White compared to other racial groups, ensuring data consistency using Stata software to convert datasets,^25^ revealed a significant difference. This subanalysis, incorporating data from three studies and five datasets, showed that White patients had a lower risk of CRT+ICI-induced pneumonitis compared to those of other racial or ethnic groups (HR = 0.78, 95% CI = 0.62–0.97, *p* = 0.03) (Figure 7C).^25^^,^^29^^,^^38^ These findings highlight the importance of considering racial differences in assessing the risk of CRT+ICI-induced pneumonitis and suggest potential underlying biological or socioeconomic factors influencing this adverse event.

#### Programmed death-ligand 1

**Figure 8:**
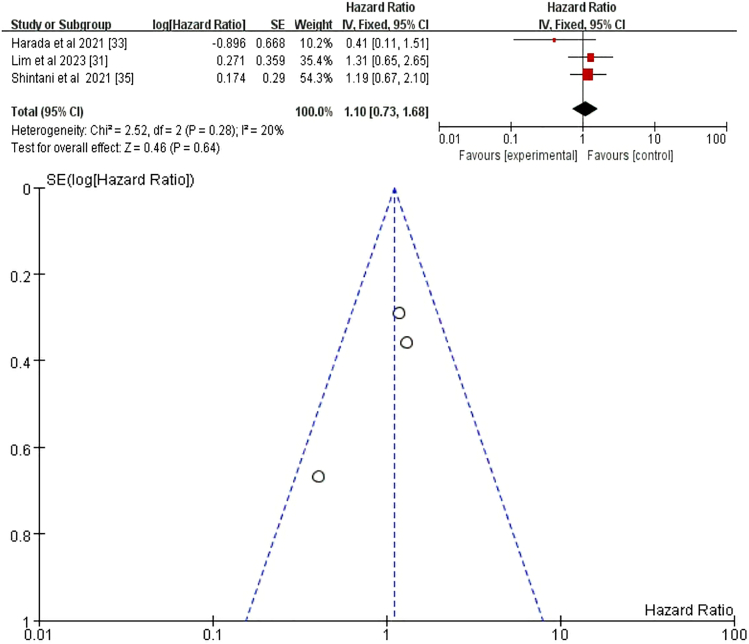
Fixed-effects model with forest plot and funnel plot for assessing the risk of pneumonia related to PD-L1 PD-L1, Programmed cell death ligand 1.

We conducted a focused analysis of three eligible studies.^31^^,^^33^^,^^35^ A meta-analysis of the data from these three studies, using fixed-effects and random-effects models, revealed consistent results (HR = 1.1, 95% CI = 0.73–1.68, *p* = 0.64) (Figure 8). These findings collectively indicate that PD-L1 expression level is not a significant risk factor for the development of pneumonitis after RCT plus ICI treatment.

#### Pulmonary comorbidity and chronic obstructive pulmonary disease

**Figure 9:**
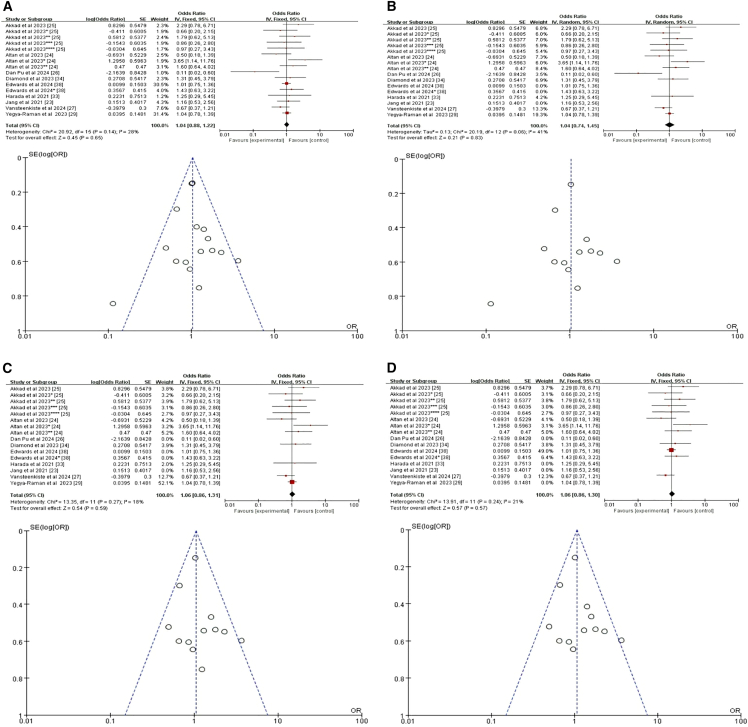
Forest plot and funnel plot of studies on pulmonary comorbidity and pneumonitis risk (A) Fixed-effects model for the risk of pneumonia related to pulmonary comorbidity. (B) Excluding two studies on treatment-unspecified pneumonitis, the random-effects model analyzed pulmonary comorbidity-related pneumonia risk. (C) Excluding one study with the greatest impact on pneumonitis, the fixed-effects model analyzed pulmonary comorbidity-related pneumonia risk. (D) Fixed-effects model for the risk of pneumonia related to COPD. COPD, chronic obstructive pulmonary disease.

We analyzed data from nine studies,^23^^,^^24^^,^^25^^,^^26^^,^^27^^,^^29^^,^^33^^,^^34^^,^^38^ which resulted in sixteen datasets due to varying reporting methods (including two datasets from one study,^38^ three from another,^24^ and five from a third^25^). Two studies conducted both univariate and multivariate analyses without differentiating treatment-induced pneumonitis, potentially introducing heterogeneity.^23^^,^^38^ An initial meta-analysis of all nine studies found no significant association between pulmonary comorbidity and risk of pneumonitis after RCT+ICI (HR = 1.04, 95% CI = 0.88–1.22, *p* = 0.65) (Figure 9A). To minimize confounding, we excluded the two studies that did not distinguish treatment-induced pneumonitis, leaving seven studies with thirteen datasets. This refined meta-analysis also showed no significant effect (HR = 1.04, 95% CI = 0.74–1.45, *p* = 0.83) (Figure 9B).^24^^,^^25^^,^^26^^,^^29^^,^^33^^,^^34^ Sensitivity analysis, excluding the study with the greatest impact,^26^ which confirmed this nonsignificant trend (HR = 1.06, 95% CI = 0.86–1.31, *p* = 0.59) (Figure 9C). Given the heterogeneity within the pulmonary comorbidity category, we conducted a subgroup analysis specifically on COPD; a common comorbidity in patients with lung cancer. This analysis, including five studies with nine datasets, revealed no significant association between COPD and the risk of pneumonitis following RCT+ICI (HR = 1.06, 95% CI = 0.86–1.30, *p* = 0.57) (Figure 9D).^24^^,^^25^^,^^27^^,^^34^^,^^38^ This underscores our primary findings and indicates that COPD, despite its clinical significance, does not independently increase the risk of pneumonitis in this treatment context.

#### Eastern cooperative oncology group performance status

**Figure 10:**
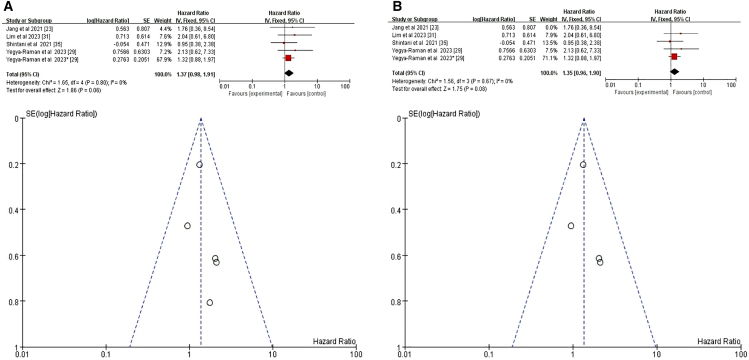
Forest plot and funnel plot of studies on ECOG and pneumonitis risk (A) Fixed-effects model for the risk of pneumonia related to ECOG. (B) Excluding two studies on treatment-unspecified pneumonitis, the fixed-effects model analyzed ECOG-related pneumonia risk. ECOG, Eastern Cooperative Oncology Group.

We analyzed data from four studies,^23^^,^^29^^,^^31^^,^^35^ yielding a total of five datasets due to one study contributing two relevant datasets on ECOG PS.^29^ One study conducted analyses without differentiating treatment-induced pneumonitis, potentially introducing heterogeneity.^23^ Initial meta-analysis incorporating all four studies showed a nonsignificant trend toward increased pneumonitis risk with poorer ECOG PS (HR = 1.37, 95% CI = 0.98–1.91, *p* = 0.06) (Figure 10A). Excluding the study^23^ that did not differentiate treatment-induced pneumonitis, the refined meta-analysis with three studies and four datasets also showed a nonsignificant association (HR = 1.35, 95% CI = 0.96–1.90, *p* = 0.08) (Figure 10B). These findings suggest that ECOG PS, while important for overall cancer prognosis, is not a significant risk factor for pneumonitis after CTR+ICI.

#### Total lung volume

**Figure 11:**
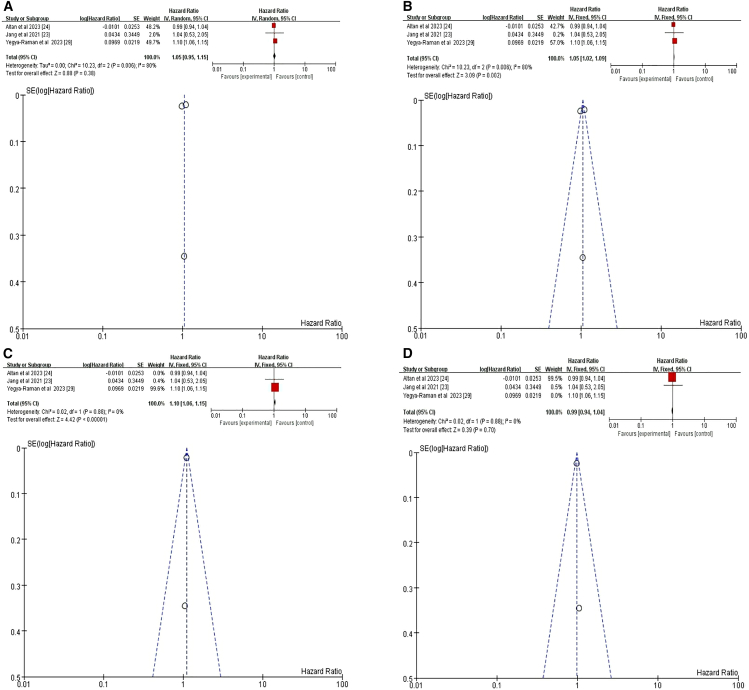
Forest plot and funnel plot of studies on TLV and pneumonitis risk (A) Random-effects model for the risk of pneumonia related to TLV. (B) Fixed-effects model for the risk of pneumonia related to TLV. (C) First subgroup: Fixed-effects model for pneumonia risk related to TLV. (D) Second subgroup: Fixed-effects model for pneumonia risk related to TLV. TLV, total lung volume.

In the primary meta-analysis examining the association between TLV and risk of pneumonitis, three studies were included.^23^^,^^24^^,^^29^ Using a random-effects model, no significant association was found (HR = 1.05, 95% CI = 0.95–1.15, *p* = 0.38) (Figure 11A). However, a fixed-effects model revealed a significant association (HR = 1.05, 95% CI = 1.02–1.09, *p* = 0.002) (Figure 11B). This discrepancy suggests potential heterogeneity among the studies. To reduce this heterogeneity, two subset analyses were conducted. The first subset, comprising two studies, showed a significant association between TLV and risk of pneumonitis (HR = 1.10, 95% CI = 1.06–1.15, *p* < 0.00001) (Figure 11C).^23^^,^^29^ In contrast, the second subset, including another pair of studies, found no significant association (HR = 0.99, 95% CI = 0.94–1.04, *p* = 0.70) (Figure 11D).^23^^,^^24^ These findings suggest that the impact of TLV on pneumonitis risk may vary based on study characteristics and methodologies. Overall, while some evidence supports an association between TLV and increased risk of pneumonitis following RCT+ICI, the results are inconsistent across studies, potentially due to differences in patient populations, treatment protocols, or pneumonia assessment criteria.

#### Mean lung dose

**Figure 12:**
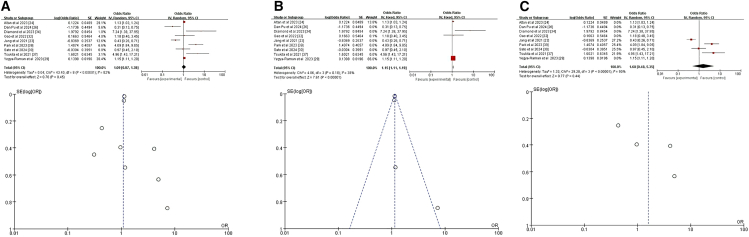
Forest plot and funnel plot of studies on MLD and pneumonitis risk (A) Random-effects model for the risk of pneumonia related to MLD. (B) First subgroup: Fixed-effects model for pneumonia risk related to MLD. (C) Second subgroup: Random-effects model for pneumonia risk related to MLD. MLD, mean lung dose.

Nine studies were included in this specific analysis to quantify the association between MLD and pneumonitis.^23^^,^^24^^,^^26^^,^^29^^,^^30^^,^^32^^,^^34^^,^^37^^,^^39^ Notably, one study conducted both univariate and multivariate analyses on the overall pneumonitis patient cohort without distinguishing the treatment regimen.^37^ Random-effects model demonstrated a non-significant association (HR = 1.09, 95%CI = 0.87–1.38, *p* = 0.45) (Figure 12A), with substantial heterogeneity (I^2^ = 90%) suggesting variability in MLD measurement protocols across institutions. To further investigate, we conducted subgroup analyses. In a subgroup of four studies^24^^,^^29^^,^^32^^,^^34^ examining MLD before radiotherapy in Gy [MLD(pre Gy)], the random-effects model showed a significant association (95% CI = 1.11–1.19, *p* < 0.00001) (Figure 12B). Conversely, in another subgroup of four studies^23^^,^^30^^,^^37^^,^^39^ comparing low versus high MLD, the results were less conclusive under the random-effects model (HR = 1.60, 95% CI = 0.48–5.35, *p* = 0.44) (Figure 12C).

#### V5

**Figure 13:**
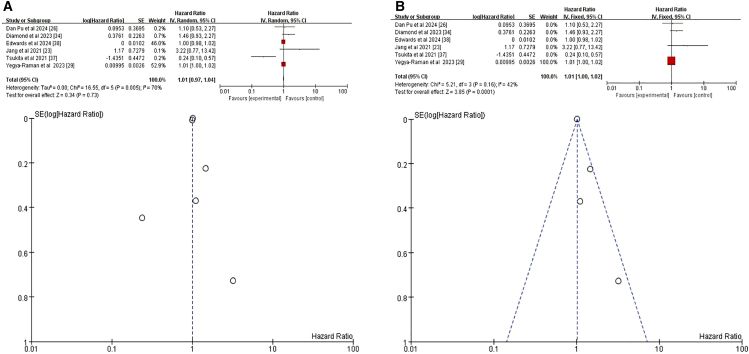
Forest plot and funnel plot of studies on V and pneumonitis risk (A) Random-effects model for the risk of pneumonia related to V. (B) Excluding two studies with the greatest impact on pneumonitis, fixed-effects model analyzed pneumonitis risk related to V. V, volume.

We conducted a comprehensive meta-analysis and systematic review, incorporating data from six studies,^23^^,^^26^^,^^29^^,^^34^^,^^37^^,^^38^ and used Stata software to ensure consistency in data metrics.^37^ Our findings revealed that there was not significant under the random-effects model (HR = 1.01, 95% CI = 0.97–1.04, *p* = 0.73), suggesting potential influence from study-specific factors (Figure 13A). To minimize confounding, we excluded two studies that did not differentiate treatment regimens causing pneumonitis.^37^^,^^38^ This adjusted analysis found a significant association between V5 and pneumonitis (HR = 1.01, 95% CI = 1.0–1.02, *p* = 0.0001) (Figure 13B). These results indicate that while V5 may modestly increase the risk of pneumonitis in patients with CTR+ICI-treated lung cancer, the association is complex and influenced by various factors.

#### V20

**Figure 14:**
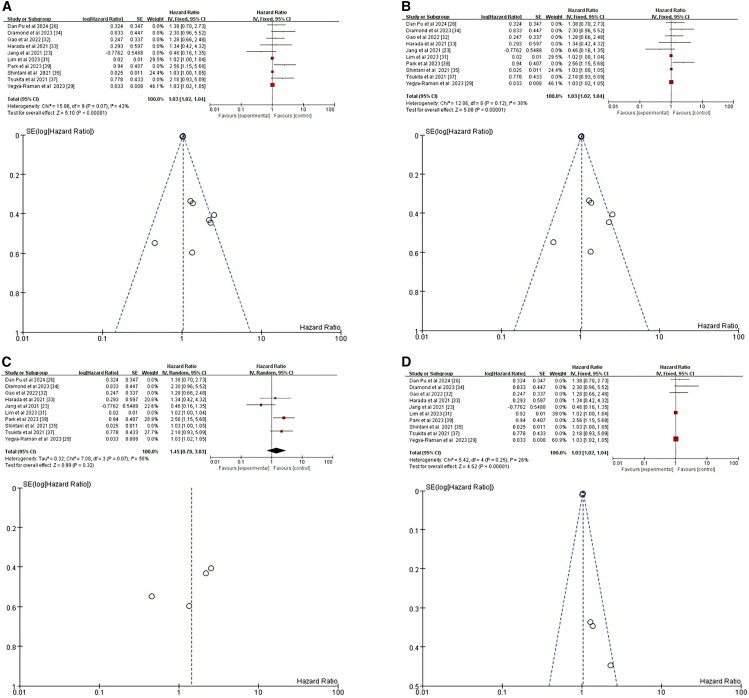
Forest plot and funnel plot of studies on V20 and pneumonitis risk (A) Fixed-effects model for the risk of pneumonia related to higher V20. (B) Excluding one study on treatment-unspecified pneumonitis, the fixed-effects model analyzed V20-related pneumonia risk. (C) Random-effects model was used for subgroup analyses comparing the risk of pneumonia between low and high V20 groups. (D) Fixed-effects model for the risk of pneumonia related to V20.

To assess the impact of V20 on the incidence of pneumonitis in patients with lung cancer undergoing thoracic CTR+ICI, we conducted a comprehensive meta-analysis and systematic review incorporating data from 10 studies.^23^^,^^26^^,^^29^^,^^31^^,^^32^^,^^33^^,^^34^^,^^35^^,^^36^^,^^37^^,^^39^ Under fixed-effects models, higher V20 values were associated with a significant but minimal increase in the risk of pneumonitis (HR = 1.03, 95% CI = 1.02–1.04, *p* < 0.00001) (Figure 14A). To minimize the impact of confounding variables, we excluded a study that did not differentiate pneumonitis cases based on treatment regimens.^37^ The adjusted analysis still found a significant association (HR = 1.03, 95% CI = 1.02–1.04, *p* < 0.00001) (Figure 14B). Subgroup analyses comparing low versus high V20 values confirmed a more pronounced risk of pneumonitis with higher V20 (HR = 1.45, 95% CI = 0.70–3.03, *p* = 0.32) (Figure 14C).^23^^,^^33^^,^^37^^,^^39^ Additionally, when focusing on studies specifically examining V20, the association between V20 and pneumonitis risk remained consistent (HR = 1.03, 95% CI = 1.02–1.04, *p* < 0.00001) (Figure 14D).^26^^,^^29^^,^^31^^,^^32^^,^^34^

#### V40

**Figure 15:**
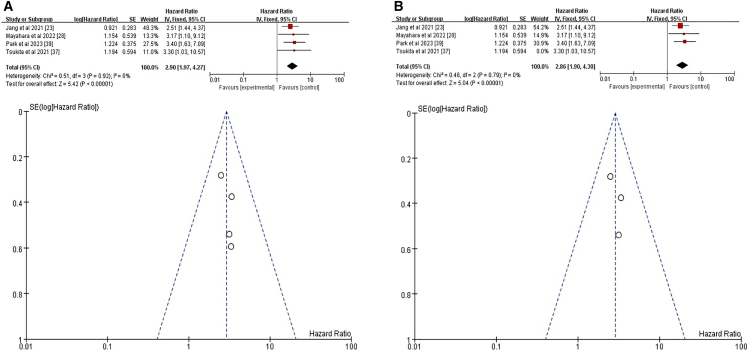
Forest plot and funnel plot of studies on V40 and pneumonitis risk (A) Fixed-effects model for the risk of pneumonia related to V40. (B) Excluding one study on treatment-unspecified pneumonitis, the random-effects model analyzed V40-related pneumonia risk.

To investigate the impact of V40 on the incidence of pneumonitis in patients with lung cancer undergoing thoracic CTR+ICI, we conducted a meticulous meta-analysis incorporating data from four studies.^23^^,^^28^^,^^37^^,^^39^ The initial meta-analysis revealed a significant association between V40 and risk of pneumonitis (HR = 2.9, 95% CI = 1.97–4.27, *p* < 0.00001 (Figure 15A). This finding indicated that an increase in V40 was associated with a substantially higher risk of developing pneumonitis following CTR+ICI. To minimize the impact of confounding variables, we excluded a study that did not differentiate pneumonitis cases based on treatment regimens.^37^ The adjusted analysis still found a significant association (HR = 2.86, 95% CI = 1.9–4.3, *p* < 0.0001) (Figure 15B). Both the initial and adjusted analyses consistently demonstrated that V40 was a significant risk factor for pneumonitis in patients with lung cancer treated with CTR+ICI.

## Discussion

In our meta-analysis of 17 studies examining pneumonitis risk factors in patients with lung cancer undergoing thoracic chemoradiotherapy and immune checkpoint inhibitors (CRT+ICI), radiation dosimetric parameters emerged as the strongest predictors. V20 and V40 demonstrated significant dose-dependent relationships with pneumonitis risk. BMI≥30 was a significant risk factor in subgroup analyses, while age exhibited a dual pattern of non-significance in pooled analysis versus elevated risk in patients ≥65 years. Notably, smoking was inversely associated with risk, and exploratory subgroup analysis suggested reduced risk in White patients. No significant associations were observed for sex, TLV, TNM stage, PD-L1 expression, ECOG score, or COPD.

Immune checkpoint inhibitors (ICIs), including anti-PD-1, anti-PD-L1, and anti-CTLA-4 antibodies, function by blocking inhibitory pathways to activate T cells, enabling tumor cell attack while increasing susceptibility to immune-related adverse events (irAEs) such as pneumonitis.^17^ Mechanistically, ICI-induced pneumonitis involves T cell–mediated tissue damage and pro-inflammatory cytokine release (e.g., TNF-α, IL-6).^14^^,^^17^ Baseline immune status, genetic factors, and prior lung disease further modulate risk,^42^ particularly with higher radiation doses.

In the treatment of patients with lung cancer, the combination of CRT + ICI has emerged as a pivotal therapeutic approach. The PACIFIC trial established consolidative durvalumab therapy combined with CRT as the standard of care for unresectable locally advanced non-small cell lung cancer (LA-NSCLC), significantly improving progression-free survival and overall survival.^10^^,^^43^ Despite survival benefits, pneumonitis remains a dose-limiting toxicity, with a grade ≥2 incidence of 18–50%^44^^,^^45^^,^^46^^,^^47^ and grade ≥3 at 4.1–8.7%.^48^^,^^49^^,^^50^^,^^51^ Although grade 3/4 pneumonitis incidence was comparable between durvalumab and placebo in PACIFIC,^43^^,^^52^ any-grade pneumonitis increased with durvalumab. Importantly, while PACIFIC mandated V20 < 35% and MLD<20 Gy,^43^ patient-level dose distribution data remain unreported, limiting correlative analyses.

While Pu et al.^26^ and Gao et al.^32^ reported non-significant associations for V5/V20/MLD under strict constraints (V20 < 30%, MLD<1500 cc), Altan et al.^24^ identified MLD as a significant risk factor (HR = 1.14, 95% CI = 1.03–1.25). These variations likely reflect heterogeneous patient cohorts, treatment protocols, and pneumonitis grading criteria.

In numerous studies, V20 has been repeatedly mentioned as an important parameter for predicting the occurrence of pneumonitis.^31^^,^^53^ Research by Shintani et al.^35^ indicated that the risk of pneumonitis increased with V20 values. Our meta-analysis also identified V20 as a significant risk factor for pneumonitis in patients with lung cancer receiving CRT combined with ICI (HR = 1.03, 95% CI = 1.00–1.06). This finding underscores the importance of V20 in predicting pneumonitis risk. However, some studies, such as cohort studies by Inoue et al.^45^ and Harada et al.,^33^ failed to find a significant association between V20 and the development of pneumonitis, potentially due to their smaller sample sizes. In contrast, studies by Mayahara^28^ and Yegya-Raman^29^ identified V20 and MLD as predictive factors for pneumonitis in patients receiving consolidative immunotherapy with ICIs, while V5 and TLD were not. Despite these differences, most studies emphasize the importance of V20 and MLD in predicting the risk of pneumonitis. The discrepancies in these findings may reflect biological variations among different study populations and differences in sensitivity to radiotherapy and immunotherapy.

Research on V40 is scarce, but recent studies suggest that V40 is also an important parameter for predicting radiation-induced pneumonitis.^54^ Studies by Lim^31^ and Gao et al.^32^ reported correlations between V40 and pneumonitis, although V40 did not emerge as an independent risk factor in multivariate analyses, but it was still associated with the occurrence of pneumonitis. Our comprehensive analysis identified V40 as a significant risk factor for pneumonitis in patients with lung cancer receiving CRT combined with ICI (HR = 2.9, 95% CI = 1.97–4.27). Mayahara’s study^28^ further underscores the importance of V40 in predicting symptomatic pneumonitis (grade ≥2).

Previous analyses have suggested that intensity-modulated radiation therapy (IMRT) may offer superior lung toxicity profiles by optimizing dose distribution.^55^ Early clinical studies suggested no exacerbated lung injury despite larger low-dose volumes.^56^ However, the consistent advantage of IMRT in reducing pneumonitis risk remains debated. While some studies, such as that by Shintani et al.,^35^ found no direct association between IMRT and pneumonitis risk, others have shown potential benefits. Stephen et al.^49^ reported lower grade 3 pneumonitis with IMRT vs. 3D-CRT (3.5% vs. 7.9%; OR = 0.41, 95% CI = 0.17–0.99), with concomitant cardiac dose reduction.^49^^,^^57^ These findings support the potential advantages of IMRT in mitigating both pneumonitis and cardiac toxicity.

Beyond radiation-related factors, patients' baseline lung conditions significantly influence the risk of pneumonitis following CRT+ICI treatment. While previous studies have identified COPD and ILD as independent risk factors for grade ≥3 pneumonitis (OR = 6.539, 95% CI = 1.953–20.705, *p* = 0.002),^26^ our meta-analysis found no significant correlation between lung comorbidities and pneumonitis incidence after CRT+ICI treatment (HR = 1.04, 95% CI = 0.88–1.22, *p* = 0.65). This trend persisted even after excluding studies that did not distinguish treatment-related pneumonitis (HR = 1.09, 95% CI = 0.87–1.36, *p* = 0.47). Similarly, subgroup analysis showed no significant association between COPD and pneumonitis risk (HR = 0.97, 95% CI = 0.77–1.21, *p* = 0.78), contradicting findings by Thomas et al.^58^ However, studies by Akkad et al.,^25^ Vansteenkiste et al.,^27^ Harada et al.,^33^ and Diamond et al.^34^ also did not find COPD to be an independent risk factor. Altan et al.^24^ identified ILD, but not COPD, as an independent risk factor for grade ≥2 pneumonitis, likely due to more severe lung tissue damage caused by ILD. ILD is a known risk factor for radiation pneumonitis in patients with LA-NSCLC^59^^,^^60^ and ICI-induced pneumonitis.^61^ In our analysis, the effect of ILD may have been diluted by study heterogeneity. Future studies should focus on ILD as a separate risk factor to better understand its contribution to pneumonitis risk.

This meta-analysis demonstrated an unexpected inverse association between smoking history and pneumonitis risk, with smokers exhibiting significantly lower incidence than non-smokers (primary analysis HR = 0.92, 95% CI = 0.88–0.97, *p* = 0.001; sensitivity analysis HR = 0.93, 95% CI = 0.88–0.97, *p* = 0.003). The protective effect was particularly pronounced for severe pneumonitis (grade ≥3: HR = 0.33, 95% CI = 0.20–0.54, *p* < 0.001),^38^ and correlated with smoking duration (per 10-pack-year increase: HR = 0.88, 95% CI = 0.80–0.97, *p* = 0.0079).^29^ These findings challenge conventional pathophysiological models and suggest smoking may attenuate pulmonary inflammatory responses to CRT+ICI.^38^ However, this does not imply that smoking is beneficial overall, as it remains a significant risk factor for lung cancer and other diseases. The observed correlation may reflect underlying patient selection biases or unacknowledged confounding factors, highlighting the need for further research to elucidate the mechanisms linking smoking to pneumonitis risk in the context of CRT+ICI treatment.

In terms of patient baseline characteristics, we conducted a detailed analysis of the risk of pneumonitis following CRT+ICI treatment. Our meta-analysis found that age was not a significant overall risk factor for pneumonitis (HR = 1.02, 95% CI = 0.99–1.05, *p* = 0.29), aligning with previous studies.^23^^,^^24^^,^^26^^,^^29^^,^^30^^,^^33^^,^^34^ However, subgroup analysis revealed a significantly increased risk in patients aged ≥65 or ≥70 years (HR = 1.39, 95% CI = 1.08–1.80, *p* = 0.01). This discovery is consistent with the findings of Tsukita et al.,^37^ who reported a significantly higher risk of pneumonitis in patients aged ≥70 years compared to those <70 years (OR = 3.63, 95% CI = 1.43–9.20, *p* = 0.0065). Given the increased risk observed in older patients, clinicians should consider this potential risk when planning treatment for patients aged ≥65 or ≥70 years.

Our meta-analysis did not identify sex as a significant risk factor for pneumonitis following CRT+ICI treatment (HR = 1.04, 95% CI = 0.85–1.27, *p* = 0.70), and this conclusion remained robust after excluding confounding factors (HR = 0.97, 95% CI = 0.77–1.22, *p* = 0.79), consistent with previous studies.^24^^,^^27^^,^^30^^,^^34^ Similarly, race was not a significant predictor of pneumonitis risk overall (HR = 0.95, 95% CI = 0.65–1.38, *p* = 0.79),^27^^,^^29^^,^^38^ although White patients had a lower risk compared to other races in subgroup analyses (HR = 0.78, 95% CI = 0.62–0.97, *p* = 0.03),^25^^,^^29^^,^^38^ aligning with higher incidence in Asian cohorts.^45^^,^^46^^,^^49^^,^^51^ BMI showed a weak but significant positive correlation with pneumonitis risk (HR = 1.03, 95% CI = 1.01–1.05, *p* = 0.001), with patients having BMI ≥30 at significantly higher risk (HR = 2.24, 95% CI = 1.12–4.48, *p* = 0.02), consistent with Akkad et al.^25^ The role of PD-L1 expression levels and TNM staging in pneumonitis risk remains unclear. Our analysis showed no significant association between PD-L1 expression and pneumonitis risk (HR = 1.10, 95% CI = 0.73–1.68, *p* = 0.64).^31^^,^^33^^,^^35^ TNM stage >IIB was also not significantly associated with pneumonitis risk (HR = 1.17, 95% CI = 0.95–1.45, *p* = 0.14),^34^^,^^37^^,^^38^ with heterogeneity limiting definitive conclusions. Subgroup differences may reflect treatment-related confounders rather than biological risk.^27^ Additionally, ECOG PS was not a significant predictor of pneumonitis risk (HR = 1.37, 95% CI = 0.98–1.91, *p* = 0.06), even after excluding studies that did not distinguish between treatment-related pneumonitis (HR = 1.35, 95% CI = 0.96–1.90, *p* = 0.08). These findings align with previous studies.^23^^,^^29^ Further research is needed to elucidate the underlying mechanisms and to identify additional risk factors that may influence the incidence of pneumonitis in patients undergoing CRT+ICI treatment.

Our study had notable strengths. Firstly, we rigorously adhered to scientific research methods, systematically and comprehensively searched multiple internationally renowned and authoritative databases such as PubMed, Web of Science, ScienceDirect, and Cochrane Library, and successfully integrated data from 17 high-quality clinical studies, ensuring the reliability and broad applicability of our findings. Secondly, this study innovatively conducted a comprehensive analysis of the relevant risk factors for CRT+ICI-induced pneumonitis in patients with lung cancer, particularly highlighting the significant impact of key indicators such as V20, V40, and BMI ≥30 on the risk of pneumonitis. Finally, the results of this study not only provide robust evidence to support current clinical decision-making but also delve into the potential impact of factors such as age, smoking history, and race on the risk of pneumonitis, providing directions for future research endeavors.

### Conclusion

In conclusion, our meta-analysis identified V20, V40, and BMI ≥30 as significant risk factors for pneumonitis in patients with lung cancer receiving CRT+ICI. Optimizing radiotherapy plans to minimize V20 and V40 exposure, implementing weight management strategies for patients with a BMI of ≥30, and enhancing monitoring for patients aged ≥65 years are recommended. While our analysis indicates a lower risk of pneumonitis in smokers, this should not affect smoking cessation protocols. Further research is needed to confirm these findings and guide clinical practice.

### Limitations of the study

This study has several inherent limitations. First, residual confounding from unmeasured variables (e.g., baseline pulmonary function, detailed immunotherapy sequencing) may persist despite multivariate adjustments. Second, heterogeneity in pneumonitis attribution methods across studies could bias risk estimates, particularly for non-dose factors such as smoking or BMI. Third, the exploratory subgroup analyses for age ≥65 years and racial differences warrant prospective validation due to potential ecological fallacies. Fourth, our inability to analyze temporal patterns of pneumonitis onset—a critical determinant of causality—restricts the mechanistic interpretation of dose-volume relationships. Finally, we did not apply established clinical significance frameworks such as Horita’s minimal clinically important difference in interpreting HR,^62^ which may limit the direct clinical applicability of our findings. These limitations necessitate cautious clinical translation of identified risk factors.

## Resource availability

### Lead contact

Further information and requests for resources and reagents should be directed to and will be fulfilled by the Lead Contact, Ting Zhang (zezht@zju.edu.cn).

### Materials availability

This study did not generate new unique reagents.

### Data and code availability

• Data: This study did not generate any new original datasets that require public deposition. The data that support the findings of this study are available from the corresponding author upon reasonable request.
• Code: No custom code was generated; all analyses were performed using publicly available software as detailed in the key resources table.
• Other items: No additional unique resources requiring specialized access procedures were generated in this study.

## Acknowledgments

No.

## Author contributions

Y.R.: conceptualization, methodology, investigation, data curation, writing– original draft, and writing – review and editing. J.H.: investigation, data curation, and writing– original draft. W.C.: methodology, investigation, and data curation. C.L.: methodology, investigation, and data curation. A.Y.: methodology, investigation, and data curation. Z.C.: methodology, investigation, and data curation. S.G.: methodology, investigation, and data curation. T.Z.: conceptualization, supervision, and writing – review and editing.

## Declaration of interests

The authors declare no competing interests.

## STAR★Methods

### Key resources table

**Table:** 

REAGENT or RESOURCE	SOURCE	IDENTIFIER
**Deposited data**		

Cochrane Library	Cochrane Library	https://www.cochranelibrary.com/
ScienceDirect	ScienceDirect	https://www.sciencedirect.com/
Web of Science	Web of Science	https://www.webofscience.com/
PubMed	PubMed	https://pubmed.ncbi.nlm.nih.gov/

### Experimental model and subject details

This systematic review and meta-analysis followed PRISMA 2020 guidelines and was registered in PROSPERO (CRD42024612910). Eligible studies enrolled adults with histologically confirmed lung cancer who received thoracic chemoradiotherapy combined with immune checkpoint inhibitors (CRT+ICI).

### Method details

#### Literature search

A comprehensive search of PubMed, Web of Science, ScienceDirect, and Cochrane Library was conducted from July to October 2024. Search terms combined keywords for pneumonitis, chemoradiotherapy, immunotherapy, and lung cancer.

#### Study selection

Randomized controlled trials and non-randomized studies were included if they: (1) enrolled patients with histologically/cytologically confirmed lung cancer; (2) administered CRT+ICI (concurrent or consolidation); and (3) reported risk factors for pneumonitis in English. Exclusion criteria: case reports, editorials, conference abstracts, incomplete data, or high risk of bias.

#### Data extraction

Two independent reviewers extracted study characteristics, patient demographics, treatment details, and pneumonitis outcomes. Discrepancies were resolved by a third reviewer.

#### Quality assessment

The Newcastle–Ottawa Scale (NOS) was used; scores ≥5 indicated moderate-to-high quality.

### Quantification and statistical analysis

Meta-analyses were performed in Stata 12.0 and Review Manager 5.4.1. Pooled HRs or ORs were calculated using inverse-variance weighted fixed-effect or random-effect models based on heterogeneity (I^2^ ≤ 50% vs. >50%; Cochran’s Q test α = 0.10). Subgroup analyses were conducted for age (≥65 years), BMI (≥30 kg/m^2^), and race (White vs. non-White). Sensitivity analyses excluded studies without clear pneumonitis attribution. Publication bias was assessed via funnel plot symmetry.
